# Spatiotemporal expression and regulation of FoxO1 in mouse uterus during peri-implantation period

**DOI:** 10.1371/journal.pone.0216814

**Published:** 2019-05-23

**Authors:** Dileyra Adiguzel, Pinar Sahin, Nilay Kuscu, Sinan Ozkavukcu, Nayce Ilayda Bektas, Ciler Celik-Ozenci

**Affiliations:** 1 Department of Histology and Embryology, School of Medicine, Akdeniz University, Campus, Antalya, Turkey; 2 Department of Obstetrics and Gynecology, Centre for Assisted Reproduction, School of Medicine, Ankara University, Ankara, Turkey; CHU Clermont-Ferrand, FRANCE

## Abstract

Recent studies indicate that FoxO1 has roles in female reproductive system, especially in maternal endometrium. Although various cellular aspects and molecular pathways have been identified, the exact molecular characteristics of embryo implantation are still not completely understood. In this study, we aimed to investigate uterine expression and regulation of FoxO1 during peri-implantation period in mice. Experimental mouse models including, normal pregnancy, pseudopregnancy, artificial decidualization, and delayed implantation and activation were performed. Our results showed that FoxO1 expression was spatiotemporal in mouse endometrial tissue throughout peri-implantation period and its expression was significantly upregulated in luminal and glandular epithelium at the time of implantation. Moreover, on day 5 morning (09:00 AM) of pregnancy, expression of FoxO1 was cytoplasmic in endometrial luminal epithelial cells where embryo homing takes place. With progressing time on day 5 evening (19:00 PM) of pregnancy FoxO1 expression was nuclear in luminal epithelium at implantation site. Pseudopregnancy and artificial decidualization models indicated that FoxO1 expression was regulated by pregnancy hormones. Delayed implantation and activation model indicated that FoxO1 expression at the time of implantation is dependent upon activation status of blastocyst due to E2 induction and uterine sensitivity to implantation. In conclusion, our findings highlight a perspective for FoxO1 expression and regulation in mouse uterus during peri-implantation period indicating that its expression is regulated by implanting embryo and pregnancy hormones.

## Introduction

Implantation is one of the crucial steps to successful pregnancy and requires reciprocal molecular dialog between the blastocyst and maternal endometrium [1–6]. Ovarian estrogen and progesterone hormones that improve blastocyst competency and uterine receptivity primarily orchestrate this molecular dialog. Uterine receptivity, namely the ‘‘window of implantation”, is a restricted period that describes the receptive state of the uterus is suitable for embryo implantation [2–3, 7–10].

In mice with the observation of a postcoital vaginal plug designated as day 1 of pregnancy, the uterus is pre-receptive on days 1–3, receptive on day 4–5 of pregnancy or pseudopregnancy. From 5^th^ day on, the uterus becomes non-receptive (refractory) to embryo implantation [3–4, 7]. Blastocyst attachment to maternal endometrium occurs in the evening of day 4 of pregnancy in mice (18:00–20:00 hr) [11–12] and becomes more prominent by the morning of day 5 of pregnancy [2, 4, 13]. During early pregnancy that involves pre-implanting embryo development, uterine receptivity, implantation and decidualization, uterus undergoes morphological, cellular, and molecular changes [1, 6]. Since many signaling molecules, morphogens, growth factors, homeotic proteins and transcription factors are involved during early pregnancy [9], it is important to understand the physiology of these processes more precisely in order to create new strategies to overcome implantation failure.

Forkhead box O1 (FoxO1) is a transcription factor and a member of the FoxO family which is the ‘O’ subclass of the large Forkhead family of proteins. Fox proteins characterized by a conserved DNA-binding domain termed the ‘forkhead box’ [14–17]. FoxO transcription factors consist of four members; FoxO1, FoxO3, FoxO4, and FoxO6 which are variably expressed in all tissues in mammals [14, 16, 18]. In the absence of growth factors, FoxOs translocate to the nucleus and upregulate a series of their target genes that regulate cellular resistance and metabolism, cell-cycle progression, oxidative stress response, and apoptosis [15, 19–21].

Recent studies indicate that FoxO1 has important roles in female reproduction system essentially by a progesterone-dependent manner [22–24]. Early studies have found that FoxO1 induces human decidualization [25]. It has been suggested that FoxO1 decides a progesterone-regulated switch at the crossroads of apoptosis and survival in human endometrial stromal cells [22]. Moreover, FoxO1 is a direct target of progesterone to inhibit endometrial epithelial cell growth [26–27]. FoxO1 is functionally required for the binding of progesterone receptor to its genomic targets [28]. It has been found that FoxO1 expression changes during the proliferative and secretory-phase in human endometrium and in mouse estrous cycle [29]. At the embryo side, FoxO1 is differentially expressed during *in vivo* pre-implantation development in mice [30]. Very recently, it has been demonstrated that FoxO1 regulates uterine epithelial integrity and progesterone receptor expression critical for embryo implantation in human and murine endometrium [31].

Based on the recent findings regarding FoxO1 transcription factor’s roles in implantation, the objective of the present study is to investigate the expression and regulation of FoxO1 in mouse uterus utilizing various experimental mouse pregnancy models and our findings highlight a perspective for FoxO1 expression and regulation in mouse uterus during peri-implantation period indicating that its expression is regulated by implanting embryo and pregnancy hormones.

## Materials and methods

### Ethics statement

Six to eight weeks old BALB/c female mice (n = 90 for all groups, 6 mice in each group) were obtained from Akdeniz University Animal Research Unit, Antalya, Turkey and the experimental protocol was approved by the Animal Care and Use Committee of Akdeniz University Faculty of Medicine **(**Number of the Ethical Approval 2015.08.02). Mice were housed in a controlled environment with a cycle of 12 L:12 D (12 h light: 12 h darkness) with *ad libitum* access to food. Mature male mice were utilized to establish the pregnancy groups. Mice were euthanized by cervical dislocation following anesthesia (Ketamine-Xylazine Mix, 0.1 ml/10g Body Weight, Intra Peritonal).

### Pregnancy and pseudopregnancy

Female mice were mated with fertile or vasectomized males of the same strain to induce pregnancy (n = 36) or pseudopregnancy (n = 30) by co-caging, respectively and day of vaginal plug was identified as pregnancy day 1. On days 1 and 4, pregnancy was confirmed by recovering embryos from the oviducts and uterus, respectively. Implantation sites on days 5 and 6 were identified by intravenous injection of 0.1 ml 1% Chicago blue in saline (Sigma, St. Louis, MO, USA) [32] and implantation sites were demarcated by discrete blue bands. The region between two implantation sites was termed as the inter-implantation sites. Mice were sacrificed to collect uteri at 09:00 hr on various days of natural pregnancy (Day 1, 4, 5, 6, and 8). Additionally, uterine tissues were collected from animals at different time points referring window of embryo implantation [Day 4 (09:00 hr), Day 4 (19:00 hr), Day 5 (09:00 hr) and Day 5 (19:00 hr)]. The entire uteri of pseudopregnant mice were collected on days 1, 2, 3, 4, 5, 6, and 8.

### Artificially induced decidualization

Decidualization was experimentally induced as described previously [33]. Briefly, artificial decidualization was induced by infusing 25 μl sesame oil (Sigma) intraluminally into one uterine horn on day 4 (09:00 hr) of pseudopregnancy while the contralateral uninjected horn served as the control (n = 18). The uteri were collected on days 5, 6, and 8 of pseudopregnancy. Decidualization was confirmed by weighing the uterine horn and by histological examination of uterine sections.

### Delayed implantation and activation

Pregnant mice were ovariectomized on day 4 of pregnancy (08:00–09:00 hr) and delayed implantation was maintained by daily injections of P4 from days 5–7. To induce blastocyst activation and implantation, P4-primed delayed mice were given an E2 injection (25 ng/mouse, s.c.) on day 7. Mice were sacrificed and uterine tissues were obtained 24 hours post-E2 injection on day 8 [34]. The implantation sites were identified by intravenous injection of Chicago blue solution. Delayed implantation was confirmed by flushing the blastocysts from the uterus. All steroids were dissolved in sesame oil.

### Immunohistochemistry

Uterine tissues fixed in 5% (v/v) formaldehyde (Merck, USA) for 24 h were dehydrated through a graded ethanol series and embedded in paraffin. Five micrometer cut uterine sections were taken. After deparaffinization and rehydration, citrate buffer (pH 6.0) was used for antigen retrieval using microwave. Subsequently, the slides were washed in PBS and endogenous peroxidase activity was blocked by 3% hydrogen peroxide in methanol for 15 mins at room temperature. After washing with PBS, the sections were treated with ultra V block (Thermo, UK; cat no:TA-125-UB,) and incubated with rabbit FoxO1 primary antibody (1:100 dilution; Cell Signaling, USA; cat no:2880S) overnight at 4°C. Next day, after washing out the primary antibody, slides were incubated for 60 min at room temperature with anti-rabbit secondary antibody (Vector, USA; BA-1000, 1/500 μL,) followed by incubation with horseradish peroxidase conjugated Streptavidin (Thermo Scientific, UK; TS-125-HR) for 30 min at room temperature. All incubation steps were performed in a humidified chamber to avoid dehydration of the slides. Positive immunoreactions were visualized with diaminobenzidine (DAB)-peroxidase substrate (Sigma; cat no:D4168) and counterstaining was performed with hematoxylin. FoxO1 expression was evaluated and photographed under a Zeiss (Oberkochen, Germany) light microscope.

### Western blotting

After homogenization and sonication, samples were centrifuged at 10,000 *g* for 10 minutes. Supernatants were collected and protein concentrations were determined using a standard bicinchoninic acid assay. Prior to electrophoresis, samples were heated for 5 min at 95°C and 50 μg protein was applied per lane. Samples subjected to SDS polyacrylamide gel electrophoresis under standard conditions were transferred onto polyvinylidene difluride (PVDF) membrane in a buffer containing 0.2 mol/L glycine, 25 mM Tris and 20% methanol, overnight at 4°C. After blocking with 5% non-fat dry milk, membranes were incubated with the following primary antibodies FoxO1 (1:300 dilution; Cell Signaling; Cat no:880S) and GAPDH (1:1000 dilution; Cell Signaling; Cat no:2118S) overnight at 4°C. After washing, membranes were incubated for 1 h at room temperature with horseradish peroxidase conjugated secondary anti-rabbit IgG diluted by 1:5000 (Vector; PI-1000). The reaction was visualized using chemiluminescence based Super Signal CL HRP Substrate System (Thermo Scientific; Cat no:34080) and the membranes were exposed to Hyperfilm. As an internal standard to confirm the equal loading of the proteins, GAPDH was loaded to the gels. ImageJ (NIH, Bethesda, MD, USA) was used to quantify the density of the western blot bands.

### H-Score analysis

Expression of FoxO1 in different compartments of endometrium including luminal epithelial cells, glandular epithelial cells, and endothelium was evaluated separately in pregnant, pseudopregnant, artificially induced decidualization, and delayed implantation and activation models. The percentage of cells at each staining intensity level was calculated, and finally an H-Score was assigned using the following formula: [1 × (% cells 1+) + 2 × (% cells 2+) + 3 × (% cells 3+)] [35]. A final score, ranging from 0 to 300, was obtained for each endometrial compartment in each group.

### Statistical analysis

Experiments were performed at least three times for each group. Data were reported as mean ± SEM. Group comparisons were made by One-Way ANOVA followed by Tukey post hoc test for parametric data and Mann-Whitney test for non-parametric data utilizing GraphPad Prism 6 software. P values <0.05 among different groups were considered statistically significant.

## Results

### FoxO1 shows distinct spatiotemporal expression during peri-implantation period in mice

**Fig 1:**
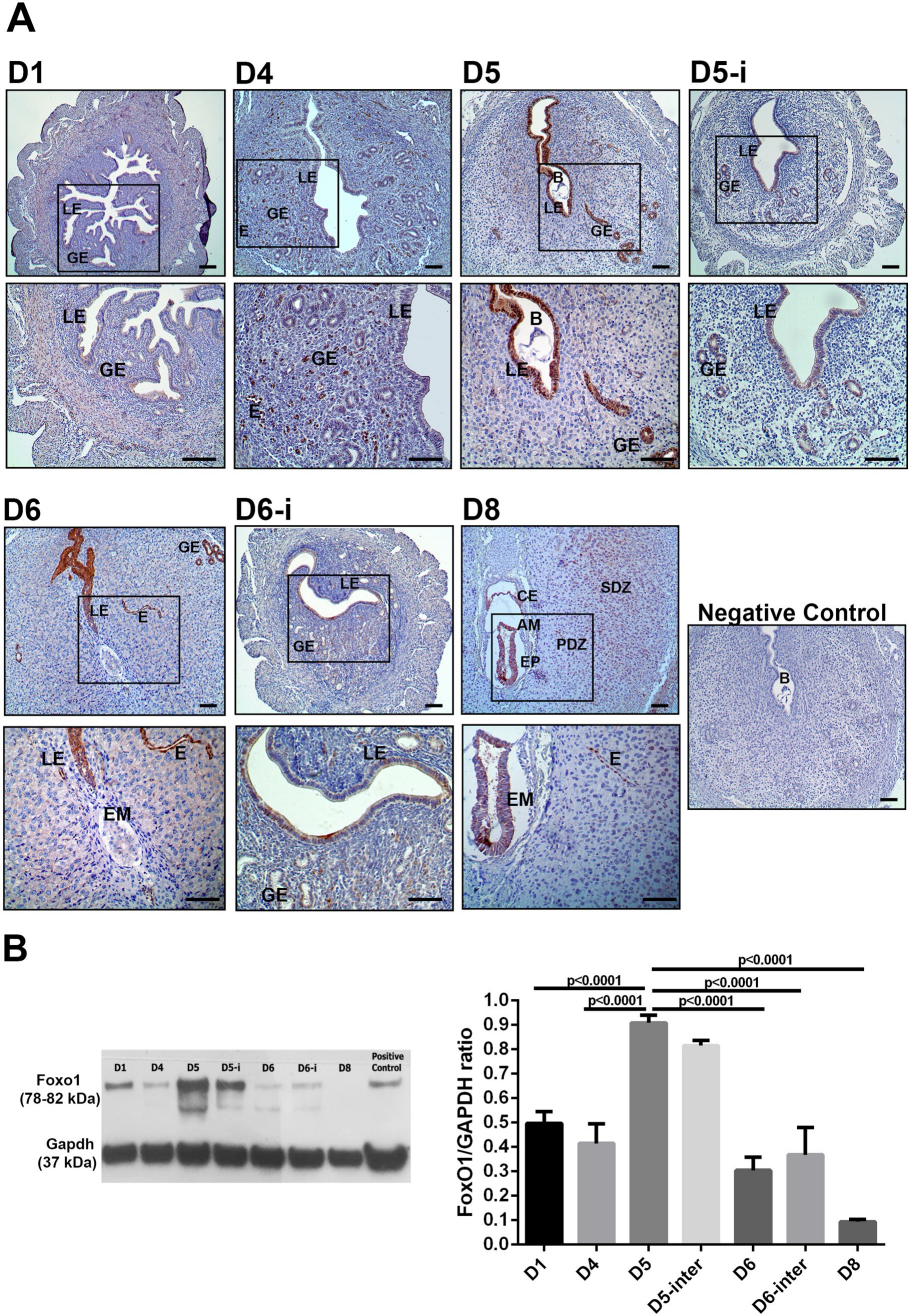
(A) Immunohistochemical staining of FoxO1 protein in the mouse uterus throughout days 1–8 of pregnancy. D5 and D6 demonstrate the implantation sites where D5-i and D6-i show inter-implantation sites on days 5 and 6 of pregnancy. Rectangles indicate higher magnifications in the lower row images. Negative control staining indicates the specifity of FoxO1 immunohistochemistry. (B) Western blot and relative densitometric analysis of FoxO1 during peri-implantation period in mice. Mouse brain tissue was used as positive control for FoxO1 expression. GAPDH levels are shown as a loading control. Experiments were repeated at least three times. LE: Luminal epithelium, GE: Glandular epithelium, E: Endothelium, B: Blastocyst, EM: Embryo, PDZ: Primary decidual zone, SDZ: Secondary decidual zone, CE: Chorionic ectoderm, AM: Amnioblasts, EP: Epiblast. NC: Negative control. Scale bars: 50 μm.

Immunohistochemistry was used to examine the spatial distribution of FoxO1 protein in mouse uterus, on days 1–8 of early pregnancy. FoxO1 exhibited distinct spatiotemporal expression during peri-implantation period in normal pregnancy. As for day 1 of pregnancy, nuclear FoxO1 expression was present in some stromal cells and its expression was weak in the cytoplasm of luminal epithelial cells (Fig 1A). On day 4 of pregnancy, nuclear FoxO1 expression was seen notably in endothelium of endometrial vessels (Fig 1A). On day 5 of pregnancy, at the time of implantation, nuclear FoxO1 expression was significantly upregulated in endometrial luminal and glandular epithelium at implantation sites (Fig 1A). Its expression was maintained in vessels. On the other hand, FoxO1 expression was weak in endometrial luminal and glandular epithelium on day 5 of pregnancy at inter-implantation sites, which is the region between the two adjacent implantation sites (Fig 1A). On day 6 of pregnancy, FoxO1 expression was maintained in regressing luminal epithelium and in endometrial vessels (Fig 1A). Conversely, its expression was weak at inter-implantation sites (Fig 1A). On day 8 of pregnancy, FoxO1 expression was present in vessels of vascular secondary decidual zone. Expression of FoxO1 was cytoplasmic in decidual cells in the secondary decidual zone on day 8 of pregnancy. Moreover, a specific cytoplasmic expression of FoxO1 was seen in epiblast, amnioblast, and chorionic ectoderm of the developing embryo (Fig 1A).

The expression pattern of FoxO1 was further verified by western blot analysis. FoxO1 expression significantly increased on day 5 of pregnancy when compared to day 1 (p<0.0001), and day 4 (p<0.0001). FoxO1 expression significantly decreased on day 6 (p<0.0001) and day 8 (p<0.0001) when compared to day 5 of pregnancy (Fig 1B).

### At the window of implantation, FoxO1 translocates to nucleus both in endometrial luminal and glandular epithelium

**Fig 2:**
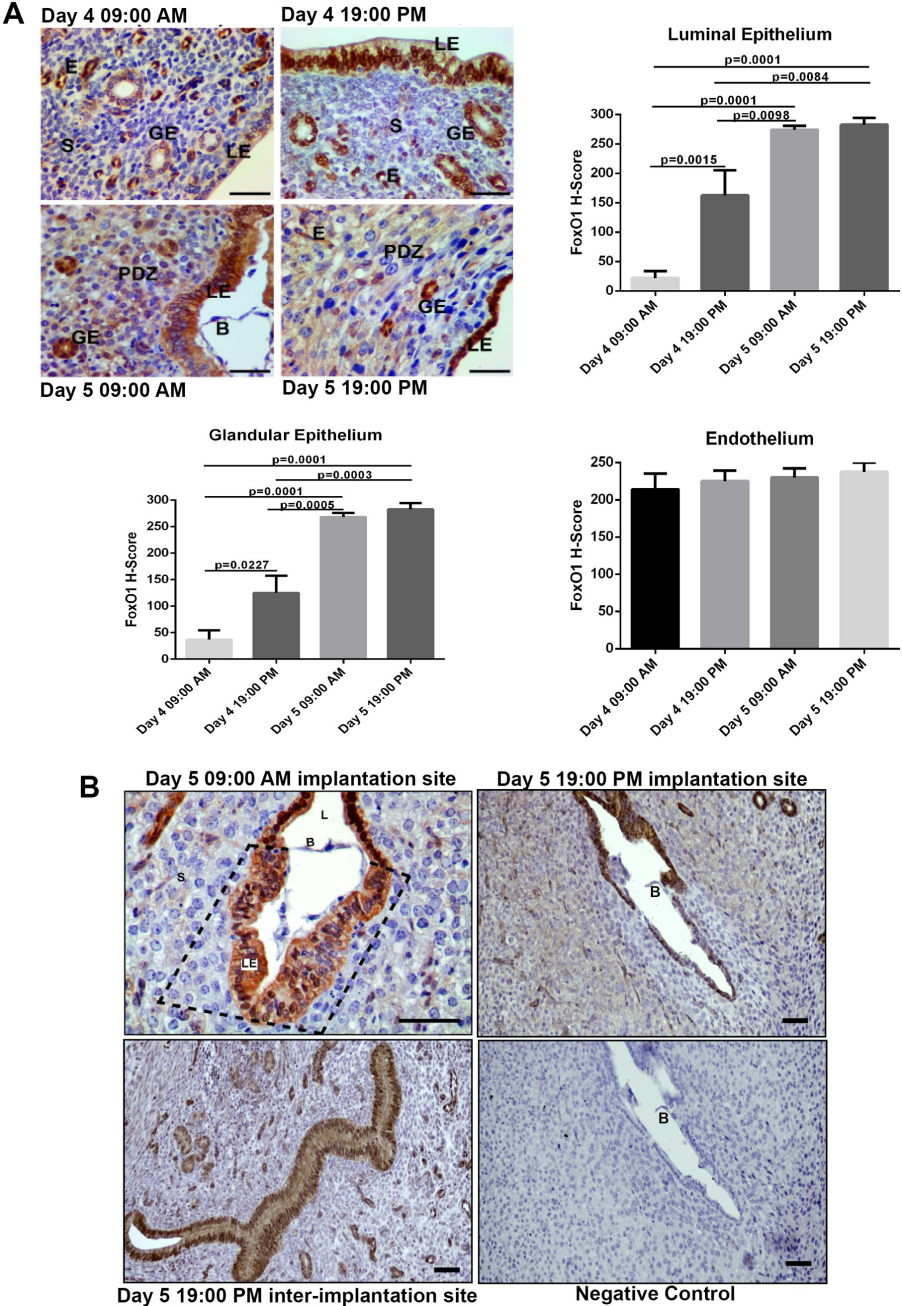
(A) Immunolocalization and H-score analyses of FoxO1 at different time intervals through days 4 and 5 of pregnancy [WOI in mice; Day 4 (09:00 AM), Day 4 (19:00 PM), Day 5 (09:00 AM) and Day 5 (19:00 PM)]. (B) Immunohistochemical expression of FoxO1 on day 5 (09:00 AM) and (19:00 PM) of pregnancy at implantation site and inter-implantation site on day 5 (19:00 PM). Note that, specifically at the region of blastocyst attachment, FoxO1 expression was cytoplasmic in luminal epithelium (dashed rectangle). LE: Luminal epithelium; GE: Glandular epithelium; S: Stroma; E: Endothelium; B: Blastocyst; PDZ: Primary decidual zone; L: Lumen. Scale bars: 50 μm.

The blastocyst attachment to maternal endometrium occurs in the evening of day 4 of pregnancy in mice (18:00–20:00 hr) [11–12]. Therefore, we evaluated FoxO1 protein expression at different time points throughout window of implantation, including early day 4 (09:00 AM), late day 4 (19:00 PM), early day 5 (09:00 AM), and late day 5 (19:00 PM). FoxO1 expression was present in the cytoplasm of both endometrial luminal and glandular epithelium on early D4 (09:00 AM), then its expression was specifically present in the nuclei of luminal and glandular cells on late D4 (19:00 PM). Nuclear expression of FoxO1 in these cells was maintained on early D5 (09:00 AM) and late D5 (19:00 PM) (Fig 2A). When we evaluated the total expression of FoxO1 in luminal epithelial, glandular and endothelial cells at time intervals we found that its expression increased significantly in luminal epithelial, glandular cells with progressing time (p = 0.0001) (Fig 2A). On the other hand, FoxO1 expression in endothelium did not differ at time intervals of window of implantation period (Fig 2A).

### On day 5 (09:00 AM) of pregnancy, expression of FoxO1 was cytoplasmic in endometrial luminal epithelial cells where embryo homing takes place

On day 5 morning (09:00 AM) of pregnancy, expression of FoxO1 was cytoplasmic in endometrial luminal epithelial cells notably only in blastocyst attachment region (dashed rectangle, Fig 2B), at embryo homing site, while its nuclear expression was still present at endometrial epithelial lining site other than surrounding the blastocyst (Fig 2B). Figure represents findings from four implantation sites of different mice (n = 4). With progressing time on day 5 evening (19:00 PM) of pregnancy FoxO1 expression was nuclear in luminal epithelium at implantation site (Fig 2B). On the other hand, at inter-implantation sites on day 5 evening (19:00 PM) of pregnancy expression of FoxO1was nuclear in luminal epithelial cells (Fig 2B).

### FoxO1 shows waxing and waning expression in individual uterine compartments through days 1–8 of pseudopregnancy

**Fig 3:**
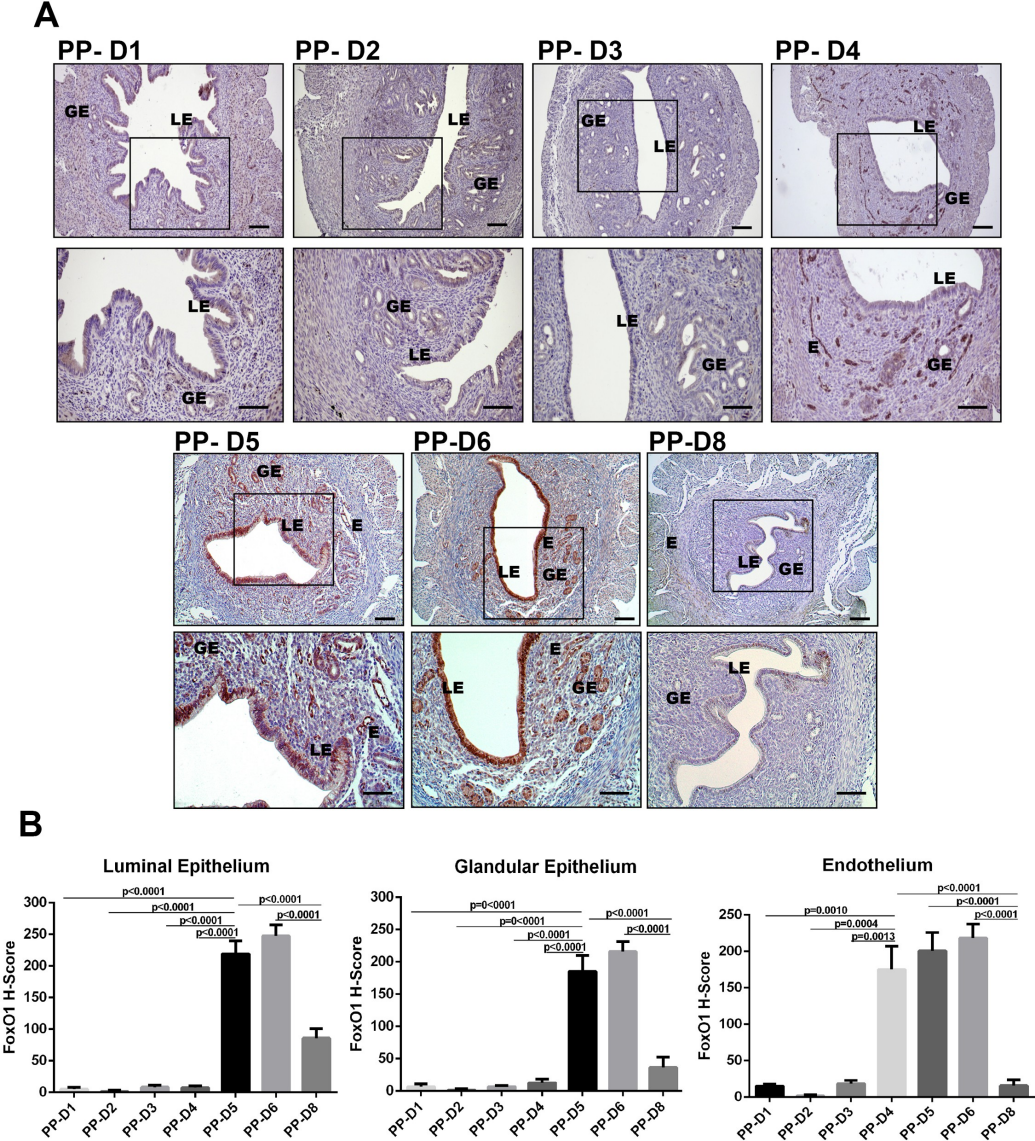
(A) Immunohistochemical expression of FoxO1 in the mouse uterus during pseudopregnancy on days 1, 2, 3, 4, 5, 6 and 8 (PP-D1, PP-D2, PP-D3, PP-D4, PP-D5, PP-D6, and PP-D8). Rectangles indicate higher magnifications in the lower row images. (B) H-score analyses of FoxO1 during pseudopregnancy uterus in mice. LE: Luminal epithelium, GE: Glandular epithelium, E: Endothelium. Scale bars: 50 μm.

To address whether FoxO1 expression was dependent on the presence of the embryo and to the altering hormonal milieu, we examined the expression of FoxO1 during pseudopregnancy. Starting from day 1 to day 4 of pseudopregnancy, FoxO1 expression was weak in the cytoplasm of luminal epithelial and glandular epithelial cells (Fig 3A). On day 4 of pseudopregnancy, expression of FoxO1 increased significantly in endothelium of endometrial vessels (p = 0.0013) (Fig 3A). On days 5 and 6 of pseudopregnancy, FoxO1 expression increased significantly when compared to days 1, 2, 3, and 4 of pseudopregnancy in luminal epithelium (p<0.0001) and in glandular epithelium (p<0.0001). Moreover, FoxO1 expression decreased significantly on day 8 when compared to days 5 and day 6 of pseudopregnancy, in luminal epithelium, glandular epithelium, and endothelium (p<0.0001) (Fig 3B). Immunohistochemistry results revealed that FoxO1 showed a waxing and waning protein expression through days 1–8 of pseudopregnancy (Fig 3A and 3B).

### Artificial stimulation of decidualization maintains FoxO1 expression on day 8

**Fig 4:**
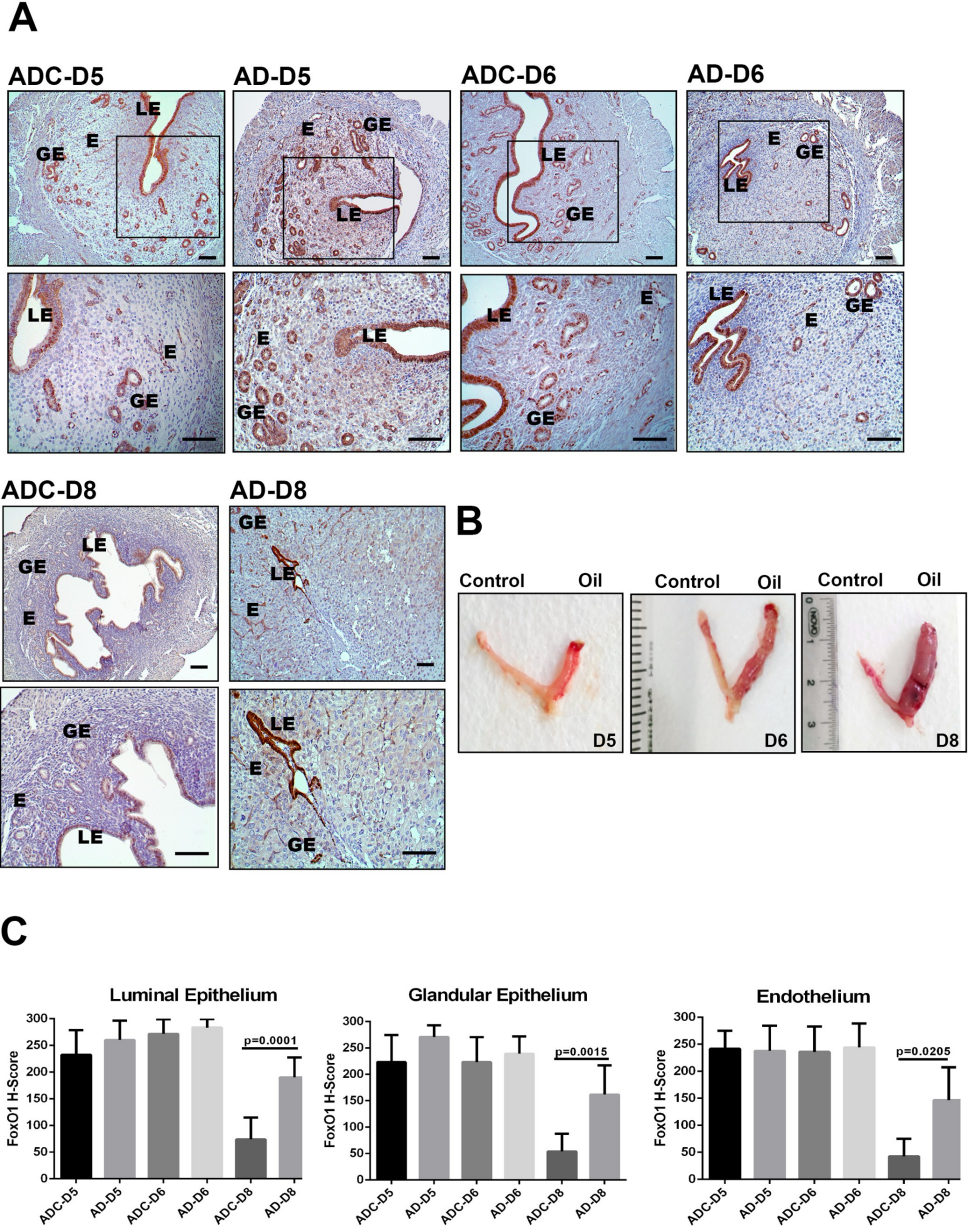
(A) FoxO1 expression in the mouse uterus under artificial decidualization on days 5, 6 and 8. ADC-D5, ADC-D6, and ADC-D8, uninjected uterine horn for control; AD-D5, AD-D6, and AD-D8, uterine horn under artificial decidualisation. Rectangles indicate higher magnifications in the lower row images. (B) A representative photo showing the deciduoma on day 5, 6 and 8 pseudopregnant mice under artificial decidualization treated with oil. (C) H-score analyses of FoxO1 in the mouse uterus under artificial decidualization. LE: Luminal epithelium, GE: Glandular epithelium, E: Endothelium. Scale bars: 50 μm.

We evaluated whether FoxO1 expression pattern changes during artificially induced decidualization, despite absence of a living embryo (Fig 4B). On days 5 and 6 of artificial decidualization, similar to normal pregnancy, FoxO1 immunolocalization was observed intensively in luminal epithelium, glandular epithelium, and endothelium of the endometrium (Fig 4A). On day 8, expression of FoxO1 significantly decreased in control horn (luminal epithelium; p = 0.0001, glandular epithelium; p = 0.0015, and endothelium; p = 0.0205) whereas its expression was maintained in artificially decidualized horn in all compartments of the endometrium when compared to days 5 and 6 (Fig 4C).

### FoxO1 expression is upregulated by activation of delayed implantation after E2 treatment

**Fig 5:**
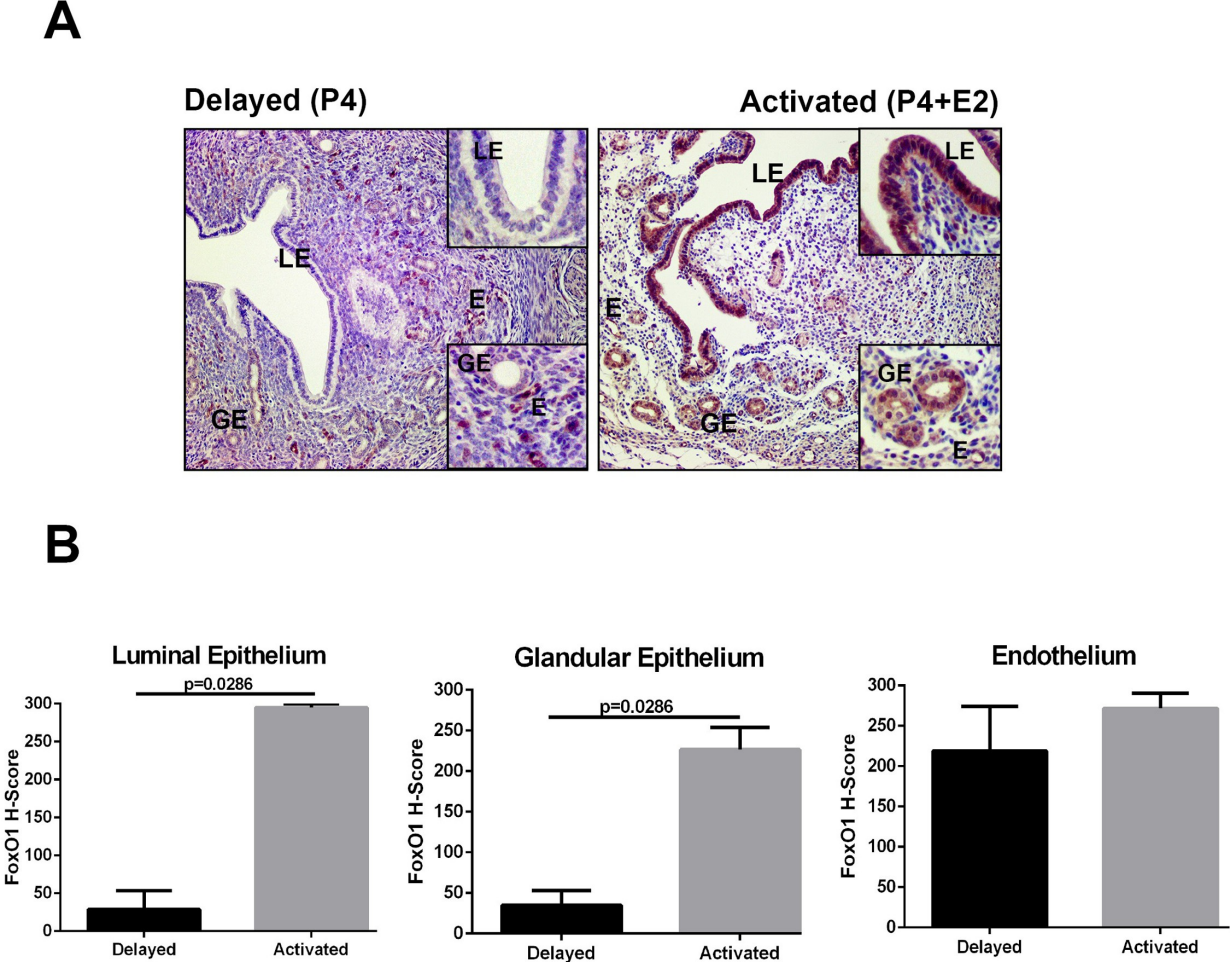
FoxO1 expression in ovariectomized, delayed implanting uteri before and after E2 activation. (A) Delayed implantation (Delayed) and activation of delayed implantation by estrogen (Activated). (B) H-score analyses of FoxO1 in the mouse uterus under delayed implantation and activation. LE: Luminal epithelium, GE: Glandular epithelium, E: Endothelium. Scale bars: 50 μm.

In order to see whether FoxO1 expression was dependent on activation status of blastocyst and uterine sensitivity to implantation, a delayed implantation model was performed. Under delayed implantation, FoxO1 protein expression was detectable only in endometrial vessels in mouse uterus (Fig 5A). When delayed implantation was terminated by E2 treatment and embryo implanted, FoxO1 expression was observed in luminal and glandular epithelium (Fig 5A). The intensity of FoxO1 staining increased significantly in luminal epithelium (p = 0.0286) and glandular epithelium (p = 0.0286) endothelium after E2 treatment when compared to the progesterone-primed delayed implantation uterus (Fig 5B).

## Discussion

The exact molecular characteristics of embryo implantation are still not completely understood. Since recent studies has indicated FoxO1’s roles in female reproductive system [22, 27–29] as it is differentially expressed during the proliferative-phase and secretory-phase in human endometrium and mouse estrous cycle [29] as well as a very recent study found that *Foxo1* ablated mice were infertile due to defective implantation and invasion [31] we investigated expression and regulation of FoxO1 during mouse peri-implantation period utilizing various experimental pregnancy models. Our results showed that FoxO1 expression was spatiotemporal in mouse endometrial tissue throughout peri-implantation period and was significantly upregulated in luminal and glandular epithelium at the time of implantation. However, its specific expression in nuclei of endometrial epithelial cells at the window of implantation decreased only in endometrial luminal epithelial cells where embryo homing takes place. Moreover, pseudopregnancy and artificial decidualization models have revealed that FoxO1 expression is regulated by pregnancy hormones. Additionally, delayed implantation and activation model designated that FoxO1 expression at the time of implantation is dependent upon activation status of blastocyst due to E2 induction and uterine sensitivity to implantation. Our findings highlight a perspective for FoxO1 expression and regulation in mouse uterus during peri-implantation period indicating that its expression is regulated not only by implanting embryo and progesterone but also with estrogen.

Mouse uterus receptivity is formed on day 4–5 of pregnancy and blastocyst attachment to maternal endometrium occurs in the evening of day 4 of pregnancy (18:00–20:00 hr) [11–12]. Since timely expression of various molecules in endometrium is important for successful embryo implantation, we investigated FoxO1 expression at different time points throughout the window of implantation. We found that FoxO1 expression became apparent firstly in the cytoplasm of endometrial epithelial and glandular cells on early D4 (09:00 hr) and then its expression was translocated to the nuclei of endometrial epithelial and glandular cells on late D4 (19:00 hr) as the uterus gains a receptive status for blastocyst attachment. FoxO proteins shuttle in and out of the nuclear compartment owing to their nuclear localization sequences and nuclear export sequences, depending on kinase cascades and its interactions with other proteins to transcriptionally regulate their downstream target genes [16, 19, 36–37]. In our study, since FoxO1 expression translocated to the nuclei of the luminal and glandular epithelium at the time of implantation it may be suggested that FoxO1 would activate or inhibit its target genes to support implantation. A recent study by Vasquez et al. analyzed the uterine transcriptome after Foxo1 ablation and found altered gene expression related to activation of cell invasion, molecular transport, apoptosis, β-catenin signaling pathway, and an increase in progesterone receptor signaling [31]. A remarkable finding of our study was that expression of nuclear FoxO1 notably decreased in endometrial luminal epithelial cells, specifically at the blastocyst attachment region, at embryo homing site at day 5 morning of pregnancy (Day 5 09:00 AM implantation site), while its nuclear expression was still present at endometrial epithelial lining without the blastocyst. On the other hand, interestingly we found that FoxO1 expression was nuclear in luminal epithelium at day 5 evening of pregnancy (Day 5 19:00 PM implantation site). Thus, we agree with the results of Vasquez et al. since they also showed a nuclear FOXO1 expression in luminal epithelium at day 5 of pregnancy (pregnancy day 0,5 = vaginal plug) [31]. In the literature of mice pregnant uterus studies, it is common to accept the pregnancy day as day 0,5 or day 1 when vaginal plug is seen. In our study, we considered pregnancy day 1 = vaginal plug. Moreover, Yingju Li et al. revealed that luminal epithelial cells in direct contact with the blastocyst are endocytosed by trophoblast cells by adopting the non-apoptotic cell-in-cell invasion process (entosis) in the absence of caspase 3 activation. Yingju Li et al. suggest that uterine luminal epithelium surrounding the blastocyst is breached on day 5 evening of pregnancy and ensuing apoptosis [38]. Hence, we believe that in our study sequestration of FOXO1 in the cytoplasm of luminal epithelium notably only in blastocyst attachment region is required for survival of luminal epithelial cells, because it prevents apoptosis during entosis. Accordingly, these results may propose a possible navigator role for FoxO1 for embryo towards the implantation site, and/or presence of a viable embryo may modify the expression pattern on its site of implantation. Altogether, the results of our study have supported and improved that FoxO1 is a novel endometrial receptivity and/or an implantation marker due to its timely and specific expression and hormonal regulation during peri-implantation period.

In mouse uterus, under the influence of pre-ovulatory ovarian estrogen, epithelial cells undergo extensive proliferation on days 1 and 2 of pregnancy. On day 3 of pregnancy, increasing progesterone levels in serum starts to inhibit estrogen-mediated epithelial proliferation and initiates stromal cell proliferation. The anti-proliferative action of progesterone in uterine epithelium is maintained during window of implantation [3, 39]. Studies have shown that FoxO1 is a direct target of progesterone to inhibit endometrial epithelial cell growth [26–27] and estradiol regulates FoxO1’s transcriptional activity in rat uterus [40]. Thus, in our study, upregulation of FoxO1 in uterine epithelium at the time of implantation might be in response to increasing progesterone secretion for the inhibition of endometrial epithelial cell proliferation and also FoxO1 expression at the time of implantation is dependent upon activation status of blastocyst with E2 treatment. We agree with the findings of Vasquez et al. indicating that FoxO1 expression is regulated by progesterone [31], moreover we found that estrogen is also another regulator of FoxO1 expression during implantation. A recent study also found that pancreatic beta-cell proliferation induced by estradiol-17beta is FoxO1 dependent and the authors propose a model of E2/ER-Foxo1 interactions effecting DNA synthesis, in beta-cells [41] Since few studies investigated the molecular mechanism regarding regulation of FoxO1 expression by estrogen so far, then topic needs further evaluation especially during embryo implantation and also in reproduction.

In the present study, we found that nuclear expression of FoxO1 by endometrial vessels was initiated from day 4 of pregnancy and maintained until the post-implantation stages. It is known that in mice, uterine expression of proangiogenic factors dramatically increases from day 4 onward [42–43]. Based on the literature knowledge, our data revealing an upregulated FoxO1 expression in uterine vessels on day 4 of pregnancy may be related to angiogenesis. At post-implantation stage, avascular and vascular decidual zones have been formed around the invading embryo to support embryo development. Stromal cells, surrounding the embryo, start to proliferate and differentiate to form an avascular primary decidual zone on the afternoon of day 5 of pregnancy while stromal cells next to the primary decidual zone form a well-vascularized secondary decidual zone on day 8 [4]. On day 8 of pregnancy, we found that cytoplasmic FoxO1 expression was present in decidual cells in secondary decidual zone. This finding is in accordance with Vasques et al. where they suggested that the sequestration of FoxO1 in the cytoplasm of decidual cells is required for survival of the decidua, because it prevents apoptosis [31]. Moreover, at post-implantation stage, a specific cytoplasmic expression of FoxO1 was seen in epiblast, amnioblast, and chorionic ectoderm of developing embryo. We have previously shown that FoxO1 is differentially expressed during *in vivo* pre-implantation embryo development in mice [30]. Thus, our present results indicate that *in vivo* FoxO1 expression at different compartments of the developing embryo is maintained after implantation.

Pseudopregnant female mice exhibit the hormonal profile of a normal pregnant female for several days after copulation. In this model, decidualization does not occur, but the hormonal milieu, including progesterone and other early pregnancy hormones, begins to differ from pregnancy after day 7 to 8 because of the absence of a developing embryo inside the uterus. Serum progesterone level is at its highest level in pseudopregnant mice on day 6 and then it gradually decreases [44–46]. We used pseudopregnant mice model to evaluate the effect of pregnancy hormones on FoxO1 expression independent of embryo presence. We found that FoxO1 showed a waxing and waning expression pattern in different uterine compartments through days 5 to 8. Similar to normal pregnancy, strong FoxO1 expression was present in luminal epithelium, glandular epithelium, and endothelium on days 5 and 6 of pseudopregnancy. Its expression decreased significantly in all uterine compartments on day 8 of pseudopregnancy suggesting that FoxO1 expression is synchronously driven by fluctuating pregnancy hormones.

The decidual response can be induced experimentally using intraluminal sesame oil injection without the presence of an embryo. The resulting structure is called deciduoma [44–45] and deciduoma, induced by mechanical stimulation in pseudopregnant mice is similar with the decidua observed in normal pregnancy. Endogenous serum progesterone level is high in deciduoma-induced pseudopregnant mice on day 6 and without exogenous supply of progesterone it gradually decreases. On day 8 of this model, deciduoma undergoes regression after a certain period [45–46]. In the present study, we established artificial decidualization model to evaluate the effect of decidualization on FoxO1 expression independent of embryo presence. We found that FoxO1 expression was maintained in luminal epithelium, glandular epithelium, and endothelium in deciduoma-induced uterine horns throughout days 5, 6, and 8. On the other hand, FoxO1 expression decreased significantly in all uterine compartments of the control horn on day 8, suggesting that mechanical induction of decidualization maintains FoxO1 expression. Altogether, our data from pseudopregnancy and artificial decidualization models has revealed that FoxO1 seems to be regulated by pregnancy hormones and essential for decidualization in mice.

The underlying mechanism by which a uterus transits from the pre-receptive to the receptive and then to the non-receptive phase is not understood well. On the other hand, delayed implantation and activation model provides valuable information for the spatio-temporal expression of an interested molecule during window of implantation in uterus. During the delayed state, the uterine responsiveness to implantation can be prolonged with continued daily P4 injections (pre-receptive phase), and readily responds to implantation with an E2 injection [47]. In this model, we found that FoxO1 expression was upregulated in the luminal epithelium and glandular epithelium after E2 treatment. Interestingly, FoxO1 expression was only present in endometrial vessels when the uterus was under delayed implantation state. These results suggest that FoxO1 expression is regulated not only by implanting embryo and progesterone but also with estrogen for uterine sensitivity to implantation.

In conclusion, our results indicate that FoxO1 is a novel endometrial receptivity and/or an implantation marker due to its timely and specific expression and hormonal regulation during peri-implantation period. Based on the results of *in vitro* and *in vivo* study series in the literature and according to our findings, we suggest that FoxO1’s role in endometrial receptivity and embryo implantation is a progesterone and estrogen dependent process in mice.
